# EFFECTIVENESS OF FRIENDLY CALLING TO IMPROVE SOCIAL ENGAGEMENT AMONG MEALS ON WHEELS CLIENTS

**DOI:** 10.1093/geroni/igae098.0190

**Published:** 2024-12-31

**Authors:** Matthew Smith, Laura Belazis, L Carter Florence

**Affiliations:** Texas A&M University, College Station, Texas, United States; Meals on Wheels America, Arlington, Virginia, United States; Meals on Wheels America, Arlington, Virginia, United States

## Abstract

Socially vulnerable older adults are at increased risk for social disconnectedness. Friendly calling, defined as regularly scheduled check-in calls by a trained staff member or volunteer, is believed to provide companionship to older adult clients and ensure they are safe. However, less is known about the elements of success for coordinated friendly calling initiatives across multiple sites. The purpose of this pilot was to assess the effectiveness of friendly calling among Meals on Wheels (MOW) clients at seven sites across the country. To collect and manage data, a technology platform designed to coordinate meal delivery to MOW clients was integrated with a friendly calling scheduling solution and a risk assessment for social disconnectedness. All friendly callers underwent extensive training about meaningful engagement and the data platform before contacting clients. The 13-item Upstream Social Interaction Risk Scale (U-SIRS-13) was used to assess the effectiveness of the 7-site pilot between January to November 2023. A total of 10,400 calls were placed to MOW clients, totaling over 114,300 minutes of talk time. Of the 316 clients receiving friendly calls, 80.4% were at risk for social disconnectedness at baseline. On average, from baseline to 3-month follow-up, MOW clients significantly reduced their social disconnectedness risk (t=-3.15, P=0.002), with 52% of participants reporting reduced social disconnectedness risk (z=-2.92, P=0.003). Findings suggest that coordinated friendly calling initiatives can reduce social disconnectedness risk among MOW clients within short durations (i.e., 3 months), which highlights the feasibility of telephone-based intervention strategies for socially vulnerable older adults.

